# Os odontoideum with “free-floating” atlantal arch causing C1-2 anterolisthesis and retrolisthesis with cervicomedullary compression

**DOI:** 10.4103/0019-5413.69316

**Published:** 2010

**Authors:** Sanjay Behari, Awadhesh Jaiswal, Arun Srivastava, Dinesh Rajput, Vijendra K Jain

**Affiliations:** Department of Neurosurgery, Sanjay Gandhi Postgraduate Institute of Medical Sciences, Lucknow – 226 014, India

**Keywords:** Atlantoaxial dislocation, craniovertebral junction, os odontoideum, posterior dislocation, surgery

## Abstract

**Background:**

Os odontoideum (OO) with C1-2 anterolisthesis and retrolisthesis may cause cervicomedullary injury both from anterior and posterior aspects. We analyzed fourteen such patients for biomechanical issues, radiological features and management of OO with free-floating atlantal arch and review pertinent literature.

**Materials and Methods:**

Fourteen patients having nonsyndromic, reducible atlantoaxial dislocation (AAD) with orthotopic OO were analyzed. During neck flexion, their C1 anterior arch-os complex displaced anteriorly relative to remnant odontoid-C2 body. The posteriorly directed hypoplastic remnant odontoid sliding below the atlas and forward translation of the C1 posterior arch caused concomitant cervicomedullary compression. During neck extension, there was retrolisthesis of the “free-floating” C1 arch-os complex into spinal canal. Spinal stenosis and lateral C1-2 facet dislocation; Klippel-Feil anomaly; and posterior circulation infarcts were also present in one patient each, respectively. Posterior C1-2 (*n*=10) or occipitocervical fusion (*n*=3) was performed in neutral position to stabilize atlantoaxial movements.

**Results:**

Follow-up (mean, 3.9 years) assessment revealed improvement in spasticity and weakness in 13 patients. One patient had neurological deterioration following C1-2 posterior sublaminar fusion, requiring its conversion to occipitocervical contoured rod fusion. One patient with posterior circulation stroke died prior to any operative intervention. Follow-up lateral view radiographs showed a bony union or a stable construct in these 13 patients.

**Conclusions:**

OO with free-floating atlantal arch may precipitate cord injury both during neck flexion and extension. This condition may be overlooked unless lateral radiographs of craniovertebral junction are undertaken in neck extension, along with the usual ones in neutral and flexed positions. Etiological factors include C1 ring-OO unrestrained movements above the hypoplastic odontoid; upward pull on OO by alar and apical ligaments; lax C1-2 facet joint ligaments; and congenital presence of horizontal facet joint surfaces that facilitates C1-2 translation.

## INTRODUCTION

Os odontoideum (OO) is a rounded, corticated and smooth-marginated ossicle clearly separated from base of the odontoid process.^1^–^3^ An “orthotopic” OO is closely approximated to C1 anterior arch and moves in unison with it, and a “dystopic” one is intimately related to the clivus.^3^–^5^ The presence of OO commonly leads to reducible atlantoaxial dislocation (AAD);^3^,^5^–^7^ its association, however, with “free-floating” atlantal arch that causes anterior and posterior dislocation is a rare phenomenon.^1^,^6^,^8^,^9^ Excessive mobility at the C1-2 joints has the potential to cause cervicomedullary injury both from the anterior and posterior aspects, both in flexion and extension movements of the neck.^6^ Free-floating atlantal arch may be missed due to lack of awareness or because investigations directed at diagnosing it are often not carried out. Whenever OO and C1-2 anterolisthesis and retrolisthesis are present, their specific and unique management issues require adequate and timely intervention. We attempt to study the biomechanical issues, radiological features and management of OO with free-floating atlantal arch and review pertinent literature.

## MATERIALS AND METHODS

Fourteen patients (male-female ratio, 10:4; mean age, 17±5.6 years) having reducible AAD with OO were included in this study. Diagnosis of OO was based upon the presence of a prominent C1 anterior arch associated with a rounded, uniformly symmetrical and well-corticated ossicle constituting OO situated well above the remnant odontoid with the presence of smooth bony intervening margins between them.^2^,^4^,^5^ None of the patients had a syndromic AAD (Down’s syndrome, Morquio’s syndrome or metatropic dysplasia).^10^

**Figure 1:**
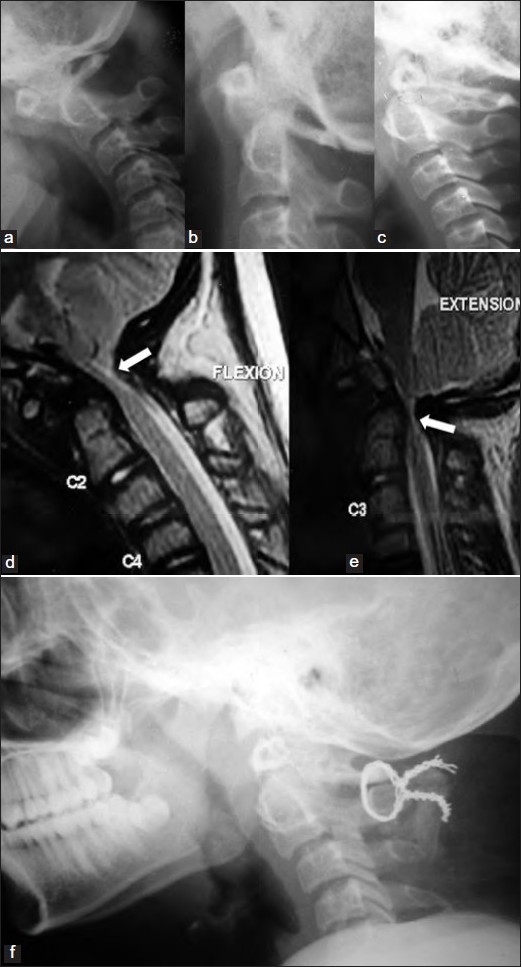
Plain lateral radiographs of craniovertebral junction in flexion (a), neutral position (b) and extension (c) of neck showing cervicomedullary compression in both flexion and extension. T2-weighted sagittal MR images in flexion (d) extension (e) showing cervicomedullary compression (arrows) in both flexion and extension. Postoperative radiograph (f) showing C1-2 sublaminar fusion with mild OO retrolisthesis

The patients were diagnosed with lateral plain radiographs of cervical spine in neutral, flexed and extended neck positions. These images were supplemented with multiplanar computed tomographic (CT) and/ or magnetic resonance (MR) images. In all patients, during neck flexion, a C1-2 anterolisthesis was evident. The C1 anterior arch-os complex moved forwards, 4.5 mm or more, relative to anterior margin of the attached part of odontoid process–body of axis.^4^,^5^,^10^–^15^ The posteriorly directed hypoplastic remnant of attached odontoid sliding below the C1 arch and the forward translation of the posterior arch of the atlas caused a concomitant cervicomedullary compression. During neutral position of the neck, the anterior C1 arch-os complex resumed its alignment with remnant odontoid process-body of axis, relieving the cervicomedullary compression. During extended position of the neck (where the C1-2 alignment in reduced position is maintained in the usual reducible AAD), in these patients, the free-floating C1 arch-os complex had a translational backward movement into the spinal canal. This compressed the cervicomedullary junction from the anterior aspect [Figure 1a–f].

**Figure 2:**
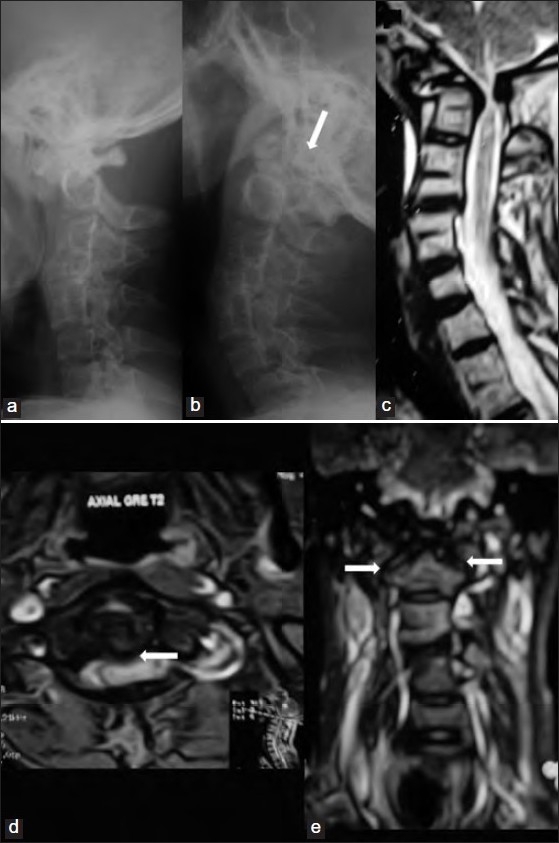
Lateral radiograph of craniovertebral junction in flexion (a) extension (b) and sagittal T2-weighted MR image (c) showing OO with Klippel-Feil anomaly (C3-4, C5-6 and thoracic 2-3 vertebral fusions) with retrolisthesis of C1 arch-os odontoideum complex (arrow) during neck extension (b). T2-weighted axial MR image (d) showing significant cord compression (arrow) with T2 hyperintense changes; and coronal image (e) showing the nearly symmetrical facet joint surfaces (arrows)

The patients had symmetrical facet joints and well-formed C1 posterior arch. In one patient, C1-2 facet joint lateral dislocation coexisted, highlighting the excessive C1-2 mobility. One patient also had Klippel-Feil anomaly with C3-4, C5-6 and thoracic 2-3 vertebral body fusion. Due to abnormal mobility between the C1-2 joints, he did not complain of any restriction of neck movements prior to undergoing C1-2 posterior fusion [Figure 2a–e]. The patients (with the exception of one) presented with spastic quadriparesis. Three patients had a history of disproportionate quadriparesis following minor trauma that improved over time once their neck movements were stabilized. Three patients were nonambulant, with dyspnea at rest and hesitancy and precipitancy of micturition. One of them had associated hydrocephalus that had been successfully treated with a ventriculoperitoneal shunt. This patient also had mild mental retardation. One of our patients who was earlier diagnosed to be having an OO, AAD with simultaneous C1-2 anterior and posterior listhesis developed sudden cerebellar and occipital infarcts and presented with absent brainstem signs, gasping respiration and flaccid paraplegia. He was placed on ventilatory support but succumbed before operative intervention.

The patients were placed on a hard cervical collar (except the one who presented with posterior circulation stroke, and was placed on Crutchfield traction) and underwent either a posterior C1-2 (n=10)^6^,^16^–^19^ or occipitocervical fusion (n=3).^20^,^21^ The posterior fusion was performed in prone position with the neck maintained in neutral position after ensuring C1-2 joint reduction and OO-remnant odontoid and C2 body alignment using X-ray image intensifier. C1-2 fusion was performed by modified Brook’s technique^6^,^16^ using either stainless steel wires (n=6; Aesculap) or braided sof’ wire (n=4; Meditronics). Occipitocervical fusion using contoured rod (n=3)^20^,^21^ was performed in the following circumstances:

- Sublaminar C1-2 posterior arch tightening led to C1-os complex retrolisthesis into the spinal canal (n=2). Occipitocervical contoured rod helped in retaining the reduced C1-2 position by maintaining the relative distances between C1 posterior arch and C2 lamina. Strut and onlay bone grafts were placed in the resultant space between the decorticated C1-2 posterior elements.
- An associated atlantal stenosis mandated removal of the C1 posterior arch and an occipitocervical fusion (n=1). In these three patients, who underwent an occipitocervical fusion, drilling of the posterior rim of foramen magnum and the posterior arch of atlas relieved posterior thecal compression and also ensured that the loop of the sublaminar wires remained well clear of the dura at the cervicomedullary junction during tightening of the wires. Onlay split autologous rib bone grafts were placed on decorticated C1-2 posterior elements. During surgery, neck movements were stabilized using a Gardner Well’s or Crutchfield’s cervical traction with the traction weight being 5% to 7% of body weight. In three patients, fiber-optic bronchoscopic intubation was performed.

Following surgery, the patients were placed on a hard cervical collar for a minimum period of three months. They were discharged on the seventh postoperative day and followed up after six weeks and thereafter at three-monthly intervals.

## RESULTS

The mean follow-up was 3.9 years (range 4 months to 5.4 years). There was significant improvement in spasticity and weakness in 13 of the 14 patients. The three patients with advanced myelopathy improved to the status of walking without support though with residual persisting spasticity. Their respiratory difficulty and sphincteric dysfunction improved. One patient had neurological deterioration in all four limbs (grade 0-I) following posterior stabilization; and had aggravation of respiratory difficulty, for which the patient had to be ventilated for three postoperative days. He required re-surgery and conversion of C1-2 sublaminar fusion to occipitocervical contoured rod fusion. The patient with subaxial blocked vertebrae complained of neck movement restriction following C1-2 fusion. The patient who developed posterior circulation stroke died within a week following admission and was not operated keeping in view his poor neurological status. Bony union of the construct was assessed on the follow-up lateral radiograph/CT scan of the craniovertebral junction (CVJ) after a minimum period of three months. The presence of the well-visualized bone grafts traversing the length of the posterior surface of occipitocervical or C1-2 bones and maintaining a good bony contact and/ or a bony fusion signified a stable construct. At follow-up, the remaining 13 patients maintained their improved status with a stable construct.

## DISCUSSION

### Anatomical and biomechanical considerations

The presence of OO manifests in four different ways:

A: If a dystrophic OO^4^ coexists with the remnant odontoid process (attached to C2 body) in close proximity to C1 anterior arch (maintained by a competent transverse ligament), AAD does not occur. A normal C1-2 alignment is preserved despite the presence of OO.

B: In the commonly encountered situation, on neck flexion, the C1 anterior arch-OO complex undergoes anterior translational displacement relative to the attached part of odontoid-C2 body and reduces spontaneously to its anatomical position in extension. This results in reducible AAD that causes spinal canal compromise and cervicomedullary compression only during neck flexion.^6^ After ensuring proper reduction of AAD, posterior stabilization suffices in this case.

C: The C1 anterior arch-OO complex slips anterior to the remnant odontoid. The posterior arch of the atlas also follows the translational movement and slips forward. The spinal canal at C1-2 level is therefore compromised both by the posteriorly directed remnant odontoid process and C2 body from the anterior aspect of the thecal sac; and the anterior displacement of the C1 posterior arch from the posterior aspect. The wedged OO between the anterior arch of atlas and the remnant odontoid process prevents any further alterations in the relative positions of the anterior arch-os and the remnant odontoid-C2 body segments during flexion and extension movements of the neck, and an irreducible AAD results compromising the spinal canal diameter regardless of whether the neck is in flexion or extension. In this condition, transoral decompression of the anterior arch of atlas-OO and remnant odontoid complex decompresses the thecal sac from the anterior aspect. This extensive osteoligamentous excision renders the CVJ unstable making a posterior fusion mandatory.^15^,^22^–^25^

D: The final situation, which was seen in our patients, is where the anterior arch-os complex is almost “freely floating” on the axis.^1^,^6^,^8^,^9^ Thus the ring of atlas with the attached OO moves in unison freely above and completely unhampered, even during neck extension, by the apical portion of the remnant odontoid process still attached to the body of axis. During flexion of the neck, the anterior arch of the atlas-OO complex moves forwards relative to the axis. The remnant odontoid-C2 complex (that is now oriented posteriorly into the spinal canal) and the posterior arch of the atlas (that has simultaneously slipped forwards), therefore, cause cervical canal compromise. During extension of the neck, the anterior arch of the atlas-OO complex moves backwards relative to the axis, again compromising the cervical canal. Thus there is both C1-2 anterolisthesis and retrolisthesis during dynamic flexion and extension movements of the neck, respectively [Figure 1a–e].

In our study, the main reason that emerged for hypermobility of C1-2 was that the orthotopic os, intimately attached to C1 anterior arch, renders the remnant odontoid hypoplastic. The apical and alar ligaments that are attached to the OO exert an upward vector of pull displacing the latter further away from the remnant odontoid process. Thus the ring of the atlas-os complex is permitted an unrestricted movement above the remnant odontoid-body of axis. Since the majority of movement between the C1-2 vertebrae occurs at the lateral facet joints,^3^,^10^–^12^,^26^–^28^ this free movement between the two bones cannot occur without the simultaneous presence of atlantoaxial facet incompetence. There is an embryological basis for the coexistence of OO, AAD and facet incompetence.^4^,^14^ The axis vertebra is derived from the proatlas and the first and second cervical sclerotomes. Failure of the entire odontoid process (derived from the C1 sclerotome) or the apex of the odontoid process (derived from the proatlas) to unite with the remnant odontoid process or to the body of axis results in OO. The superior portion of C1 posterior arch is derived from dorsal caudal portion of the proatlas; and its inferior portion, from the first cervical spinal sclerotome. The lateral atlantal masses are derived from the dorsal caudal segment of the neural arch of proatlas. Similarly, the lamina of axis, as well as its lateral masses, is derived from the neural arch of the second spinal sclerotome.^4^,^14^ Congenital absence of posterior C2 elements has been associated with AAD and facet joint incompetence both at C1-2 and C2-3 levels.^29^ The congenital basis of AAD has also been established by a study that has shown a significantly high association between mutation in genes coding for enzymes involved in the folic acid metabolism in these patients.^30^ OO may often be associated with other congenital anomalies, like assimilation of atlas, Klippel-Feil anomaly^7^ or atlantal ring stenosis.^22^ The latter two anomalies were in fact seen in our patients with OO. Considering the common origin of OO and the facet joints, the abnormally increased translational movement between the C1-2 vertebrae in our patients, therefore, may have been due to the congenital presence of relatively flatter atlantoaxial facet joints (rather than the normal ones, which have an oblique orientation). The coexistence of laxity of C1-2 joint ligaments may also have contributed to this exaggerated mobility.^10^ This abnormal C1-2 displacement was well exemplified in one of our patients who in addition to having anteroposterior mobility also had lateral C1-2 dislocation at the level of the facet joints, resulting in the appearance of the “naked facet” sign.^8^,^31^ One school of thought has considered OO to be a disunited fracture following trauma during early childhood.^1^–^4^,^10^ In three of our patients, the quadriparesis was precipitated by trauma. In none of the cases, however, was the trauma severe enough to cause bony injury of this magnitude; there was no history of associated neck pain or neck stiffness which would have been inevitable accompaniments with significant trauma; and the radiology showed well-corticated intervening margins of OO and the remnant odontoid process in all cases.

### Management issues

**Figure 3:**
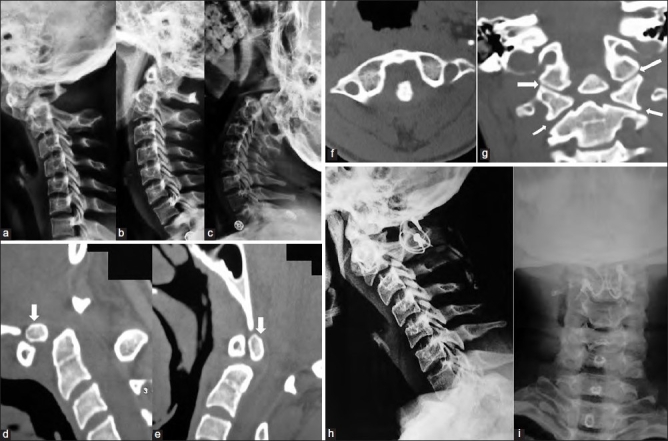
Lateral cervical spine radiograph showing AAD on neck flexion (a). C1 posterior arch is anterior to spinolaminar line and causing canal compromise. OO is impinging into the canal in neutral (b) and extended (c) neck positions. Sagittal CT in flexion (d)/ extension (e) showing anterior C1 arch-os complex (arrow) being above the tip of remnant odontoid and having anteroposterior translational movements unhindered by the latter. Axial CT (f) showing AAD. Coronal CT (g) showing OO and symmetrical facet joints (arrows). Lateral radiograph of CVJ (h) showing posterior sublaminar C1-2 fusion using so’fwires. OO and the remnant odontoid-C2 body are in alignment. C1-posterior arch and C2 lamina are closely approximated. Anteroposterior radiograph (i) shows orientation of bilaterally placed sof’wires

In contrast to patients having a conventional reducible AAD, who attain normal C1-2 alignment during neck extension, patients with free-floating atlantal arch have AAD not only during flexion but also extension of the neck [Figure 3a–f]. If the dynamic lateral radiographs of the CVJ are not undertaken in completely extended position of the neck, this entity is likely to be missed. The hard Philadelphia collar can only immobilize 40% to 60% of normal neck movements.^14^,^15^,^27^,^28^ This exaggerated mobility is therefore associated with the incessant danger of cord injury despite the presence of a protective collar. The little protection offered by the neck muscles is also eliminated after administration of muscle relaxants during general anesthesia. Intubation and positioning of the patient, once the patient has been anesthetized, have to be carried out with extreme caution, stabilizing the neck in neutral position. The proper intraoperative atlantoaxial alignment must be confirmed using X-ray image intensifier to prevent the danger of a persistent C1-2 anterolisthesis or retrolisthesis, which precipitates silent cervicomedullary damage during surgery. Preoperative application of a halo brace and its continuance during surgery maintaining proper anatomical position of the C1-2 bones would be useful in these patients.^32^ During surgery, special considerations are required while using various posterior fusion techniques. During modified Brook’s fusion,^6^,^16^,^17^ the lamina and spinous process of the axis have to be gently manipulated in an upward and forward direction towards the C1 posterior arch under image guidance. This movement angulates the remnant odontoid process attached to the axis anteriorly and prevents its backward dislocation into the spinal canal. It also prevents the posterior dislocation of the ring of atlas-os complex into the spinal canal. This optimum position is maintained by sublaminar wiring and onlay and strut bone grafts. In three patients, occipitocervical contoured rod was placed with onlay autologous rib graft stabilization.^20^,^21^,^33^ While using this method, care has to be taken to ensure that the metal rod is adequately contoured and fixed in situ under fluoroscopic guidance. The occipitoatlantal complex should be aligned with the axial and subaxial segments with no residual translational, rotational or transverse dislocation that may precipitate postoperative torticollis or neck tilt. The symmetrical C1-2 facet joints in all our patients would also permit the use of the biomechanically stable C1-2 Magerl’s transarticular fusion,^34^,^35^ Goel’s lateral mass stabilization with or without facet joint spacers^36^,^37^ or occipitocervical lateral mass fusion.^4^,^28^,^38^ In the latter techniques, image-guided restoration of proper C1-2 reduction and alignment must be ensured lest one converts a reducible AAD into an irreducible one. We admit that the stabilization achieved with C1-2 sublaminar fusion and with occipitocervical contoured rod fusion is not as biomechanically strong as it is with transarticular and lateral mass rods and plates. In the majority of our patients, the posterior arch of the atlas was well formed. The AAD was reducible and so adequate bony contact between posterior arch of atlas and the lamina of axis was easily attainable following sublaminar posterior stabilization. Onlay bone grafts on the decorticated occiput, atlas and axis ensured an adequate bony fusion while the patient was maintained on a hard cervical collar for at least three months (or until bony fusion was visualized on radiographs/ sagittal reconstructed images of CT scan of the patient). The technique was economical, simple to perform (even with coexisting cervical scoliosis or asymmetrical facet joints), effective and required the least operative time amongst all the prevalent posterior fusion techniques. Once an adequate canal diameter had been achieved following the intraoperative reduction of AAD, the sublaminar wiring could be safely performed without causing thecal sac compression.

### Unique problems

While managing this special subset of patients, we encountered certain unique problems. One of our patients presented in an unconscious state with flaccid quadriplegia, compromised respiration and absence of brainstem signs. His CT scan revealed infarcts in the posterior circulation territory. In view of his poor neurological status, no surgical intervention was attempted in his case. Several studies have demonstrated abnormalities in the course of vertebral artery and its occlusion in patients with AAD.^11^,^26^,^39^,^40^–^43^ There was no cardiac illness or any sign of atherosclerotic disease in our patient. The abnormal C1-2 mobility perhaps resulted in spontaneous dissection or thrombosis of the dominant vertebral artery without adequate hemodynamic support from the contralateral vertebral artery or the anterior circulation (via the posterior communicating arteries), which led to brainstem perforator compromise.^42^,^43^ In our patient, the extremely poor neurological status prevented us from performing an angiogram or undertaking any operative intervention. Another patient who had C1-2 “hypermobility” and Klippel-Feil syndrome with the presence of C3-4 and C5-6 blocked vertebrae was troubled by the significant restriction of lateral movements at the neck once his C1-2 joints had been stabilized [Figure 2a–c]. Approximately 37% to 47% of axial rotation of the neck coupled with minor anteroposterior translation normally occurs at the atlantoaxial joints.^4^,^10^,^27^,^28^ The subaxial spine contributes to rest of the neck rotation. In our case, the exaggerated C1-2 joint mobility was compensating for the restriction of rotation of the subaxial spine due to the two-level (4-segment) blocked cervical vertebrae. The C1-2 stabilization hampered this compensation and caused neck movement restriction. Finally, the coexistence of OO, atlantal stenosis and anteroposterior and lateral AAD in one of our patients was a unique constellation of anomalies.^8^ In this situation, the atlantal stenosis was addressed by removal of C1 posterior arch and performing an occipito-axial stabilization.^8^,^11^,^44^,^45^ Passage of sublaminar wire in the presence of atlantal stenosis, AAD and orthotopic OO would have been disastrous.^9^,^10^,^46^

The majority of our patients with free-floating atlantal arch made significant recovery, probably because of relatively early intervention prior to severe and sustained cervicomedullary injury.

## CONCLUSIONS

OO with free-floating atlantal arch may cause cervicomedullary injury both during flexion and extension of the neck. This condition is likely to be overlooked unless dynamic lateral radiographs of the CVJ in extended position of the neck are carefully evaluated along with the usual ones that are undertaken in neutral and flexed neck positions.

## References

[1] Fielding JW, Hensinger RN, Hawkins RJ (1980). Os Odontoideum. J Bone Joint Surg Am.

[2] Holt RG, Helms CA, Munk PL, Gillespy T III (1989). Hypertrophy of C-1 arch: Useful sign to distinguish os odontoideum from acute dens fracture. Radiology.

[3] Menezes AH, Marlin AE (1988). Os odontoideum: Pathogenesis, dynamics and management. Concepts in Pediatric Neurosurgery.

[4] Van Gilder JC, Menezes AH, Youmans JR (2005). Anomalies of the craniovertebral junction. Neurological Surgery.

[5] Vargas TM, Rybicki FJ, Ledbetter SM, MacKenzie JD (2005). Atlantoaxial instability associated with an orthotopic os odontoideum: A multimodality imaging assessment. Emerg Radiol.

[6] Behari S, Bhargava V, Nayak S, Kirankumar MV, Banerji D, Chhabra DK (2002). Congenital reducible atlantoaxial dislocation: Classification and surgical considerations. Acta Neurochir (Wien).

[7] Sherk HH, Dawoud S (1981). Congenital os odontoideum with Klippel-Feil anomaly and fatal atlanto-axial instability. Report of a case. Spine.

[8] Das RK, Behari S, Shinghal N, Jaiswal AK, Mahapatra AK (2007). Hydrocephalus, mental retardation, with “hypermobile” atlantoaxial dislocation due to os odontoideum, and atlantal stenosis causing compressive myelopathy. J Pediatr Neurosc.

[9] Shirasaki N, Okada K, Oka S, Hosona N, Yonenobu K, Ono K (1991). Os odontoideum with posterior atlantoaxial instability. Spine.

[10] Salunke P, Behari S, Sharma MS, Jaiswal AS, Jain VK (2006). Pediatric congenital atlantoaxial dislocation: Focusing on the differences between the irreducible and reducible varieties. J Neurosurg.

[11] Hodak JA, Mamourian A, Dean BL, Dickman CA, Spetzler RF, Sonntag VK (1998). Radiologic evaluation of the craniovertebral junction. Surgery of the craniovertebral junction.

[12] Jain VK, Behari S (2002). Management of congenital atlanto-axial dislocation: Some lessons learnt. Neurol India.

[13] Locke GR, Gardner JI, Van Epps EF (1966). Atlas-dens interval (ADI) in children: A survey based on 200 normal cervical spine. Am J Roentgenol.

[14] Menezes AH, Dickman CA, Spetzler RF, Sonntag VK (1998). Surgery of the Craniovertebral Junction. Embryology, development, and classification of disorders of the craniovertebral junction.

[15] Menezes AH, VanGilder JC, Graf CJ, McDonnell DE (1980). Craniocervical anomalies. A comprehensive surgical approach. J Neurosurg.

[16] Brooks AL, Jenkins EB (1978). Atlanto-axial arthrodesis by the wedge compression method. J Bone Joint Surg Am.

[17] Crockard A (1994). Evaluation of spinal laminar fixation by a new, flexible stainless steel cable(Sof’wire): Early results. Neurosurgery.

[18] Jain VK, Behari S, Wilkins R, Rengachary S (1997). Neurosurgical Operative atlas. Posterior occipitoaxial fusion for atlantoaxial dislocation associated with occipitalized atlas.

[19] Jain VK, Banerji D, Mittal P, Behari S, Acharya R, Chhabra DK (1996). Posterior occipital-axis fusion for atlanto-axial dislocation associated with occipitalized atlas. J Neurosurg.

[20] Kalra S, Behari S, Jaiswal AK, Jain VK (2007). Occipitocervical contoured rod stabilization: Does it still have a role amidst modern stabilization techniques?. Neurol India.

[21] Ransford AO, Crockard HA, Pozo JL, Thomas NP, Nelson IW (1986). Craniocervical instability treated by contoured loop fixation. J Bone Joint Surg Br.

[22] Crockard HA, Calder I, Ransford AO (1990). One-stage transoral decompression and posterior fixation in rheumatoid atlanto-axial subluxation. J Bone Joint Surg Br.

[23] Di Lorenzo N (1992). Craniocervical junction malformation treated by transoral approach. A survey of 25 cases with emphasis on postoperative instability and outcome. Acta Neurochir (Wien).

[24] Jain VK, Behari S, Banerji D, Bhargava V, Chhabra DK (1999). Transoral decompression for craniovertebral osseous anomalies: Perioperative management dilemmas. Neurol India.

[25] Tuite GF, Veres R, Crockard R, Sell D (1996). Pediatric transoral surgery: Indications, complications, and long-term outcome. J Neurosurg.

[26] Dumas JL, Salama J, Dreyfus P, Thoreux P, Goldlust D, Chevrel JP (1996). Magnetic resonance angiographic analysis of atlanto axial rotation: Anatomic bases of compression of the vertebral arteries. Surg Radiol Anat.

[27] Pang D, Li V (2004). Atlantoaxial rotatory fixation: Part 1--Biomechanics of normal rotation at the atlantoaxial joint in children. Neurosurgery.

[28] White AA, Panjabi MM (1978). The clinical biomechanics of the occipitoatlantoaxial complex. Orthop Clin N Am.

[29] Behari S, Kiran Kumar MV, Banerji D, Chhabra DK, Jain VK (2004). Atlantoaxial dislocation associated with the maldevelopment of the posterior neural arch of axis causing compressive myelopathy. Neurol India.

[30] Pradhan M, Behari S, Kalra SK, Das V, Agarwal S, Jain VK (2007). Association of methylenetetrahydrofolate reductase (MTHFR) genetic polymorphisms with atlantoaxial dislocation. J Neurosurg Spine.

[31] Behari S, Jain VK, Phadke RV, Banerji D, Kathuria M, Chhabra DK (2000). C1-2 rotary subluxation following posterior stabilization for congenital atlantoaxial dislocation. Neurol India.

[32] Nannapaneni R, Behari S, Todd NV (2005). Surgical outcome in rheumatoid Ranawat Class III b myelopathy. Neurosurgery.

[33] Fehlings MG, Errico T, Cooper P, Benjamin V, DiBartolo T (1993). Occipitocervical fusion with a five-millimeter malleable rod and segmental fixation. Neurosurgery.

[34] Magerl F, Seeman PS, Kehr P, Weidner A (1987). Stable posterior fusion of the atlas and axis by transarticular screw fixation. Cervical spine I.

[35] Stillerman CB, Wilson JA (2001). Atlanto-axial stabilization with posterior transarticular screw fixation: Technical description and report of 22 cases. Spine.

[36] Goel A (2004). Treatment of basilar invagination by atlantoaxial joint distraction and direct lateral mass fixation. J Neurosurg Spine.

[37] Goel A, Laheri V (1994). Plate and screw fixation for atlanto-axial subluxation. Acta Neurochir (Wien).

[38] Grob D, Dvorak J, Panjabi M, Froehlich M, Hayek J (1976). Posterior occipitocervical fusion. A preliminary report of a new technique. Spine (Phila Pa.

[39] Barton JW, Margolis MT (1975). Rotational obstruction of the vertebral artery at the atlanto-axial joint. Neuroradiology.

[40] Bhatnagar M, Sponseller PD, Carol C, Tolo VT (1991). Pediatric atlanto axial instability presenting as cerebellar and cerebral infarct. J Pediatr Orthop.

[41] Kuether TA, Nesbit GM, Clark GM, Barnwell SL (1997). Rotational vertebral artery occlusion: A mechanism of vertebrobasilar insufficiency. Neurosurgery.

[42] Puca A, Scogna A, Rollo M (2000). Craniovertebral junction malformation and rotational occlusion of the vertebral artery. Br J Neurosurg.

[43] Sawlani V, Behari S, Jain VK, Phadke RV (2006). Stretched loop sign of vertebral artery: A predictor of vertebrobasilar insufficiency in atlantoaxial dislocation. Surg Neurol.

[44] Komatsu Y, Shibata T, Yasuda S, Ono Y, Nose T (1993). Atlas hypoplasia as a cause of high cervical myelopathy. Case report. J Neurosurg.

[45] May D, Jenny B, Faundez A (2001). Cervical cord compression due to a hypoplastic atlas. Case report. J Neurosurg.

[46] Geremia GK, Kim KS, Cerullo L, Calenoff L (1985). Complications of sublaminar wiring. Surg Neurol.

